# Supplementary material for: New Fluorophore and Its Applications in Visualizing Polystyrene Nanoplastics in Bean Sprouts and HeLa Cells
Source: Molecules. 2023 Oct 15;28(20):7102. doi: 10.3390/molecules28207102 (PMC10609485; doi:10.3390/molecules28207102)
Supplement: Supplementary file 1 [file molecules-28-07102-s001.zip › Supplementary Materials.pdf]

SUPPLEMENTARY DATA 1

New Fluorophore and its Applications in Visualizing Polystyrene Nanoplastics in Bean Sprouts and HeLa Cells

Guo-wen Xing[1*] Jerry Gao[2], Heng Wang[2], Yi-chen Liu[1]

1. College of Chemistry, Beijing Normal University, Beijing, 100875, China. E-mail: gwxing@bnu.edu.cn
2. Beijing No. 80 High School, Beijing, 100102, China

（1）Density functional theory (DFT) calculations

Supplementary Data.

Calculation 1

TPEF HOMOLUMO

Method: #p opt freq 6-311+g(d,p) pop=full gfinput

TPEF -2155.640432 a.u. (zero imaginary freq)

- Calculation Type: FREQ
- Calculation Method: RB3LYP
- Basis Set: 6-311+G(d,p)
- Charge: 1
- Spin: Singlet
- E(RB3LYP): -2155.6404 Hartree
- RMS Gradient Norm: 4.00e-06 Hartree/Bohr
- Imaginary Freq: 0
- Dipole Moment: 14.115731 Debye
- Polarizability (α): 726.37401 a.u.

The coordinates of the optimized structures

C	-1.71373100	-0.11737900	0.05283300
C	-2.53255700	-1.26927100	-0.00274300
C	-3.94575000	-1.12406300	-0.02364700
O	-4.50711700	0.11805500	0.00725800
C	-3.74737700	1.24858900	0.05680700
C	-2.32803300	1.15390500	0.08466400
C	-2.04108400	-2.60020600	-0.08302200
C	-2.86365800	-3.69233000	-0.12923600
C	-4.29372100	-3.50619500	-0.13057800
C	-4.82888100	-2.18910600	-0.09061400
C	-4.43756200	2.44821500	0.07812200
C	-3.68781600	3.65518800	0.13352900
C	-2.24944300	3.59626100	0.22249100
C	-1.61986100	2.38387100	0.18715100
N	-4.31659200	4.85945900	0.11713600
C	-3.60011800	6.14294500	0.05217200
C	-2.16730900	5.99176400	-0.43747500
C	-1.47352800	4.88351900	0.35455400
C	-2.28531200	-5.08958100	-0.16352500
C	-3.24828000	-6.06676500	-0.83322200
C	-4.61415400	-5.96286900	-0.16985600
N	-5.12343600	-4.58543800	-0.19020900
C	-6.57350000	-4.42899800	-0.03217600

C	-7.06357900	-3.15145300	-0.69921400
C	-6.32160900	-1.94465600	-0.12724800
C	-5.94765000	2.48570200	0.05493500
C	-6.45285000	3.75995800	0.73176300
C	-5.78066000	4.97895600	0.11875700
C	-0.23479300	-0.24077400	0.07637500
C	0.54439900	0.27453300	-0.96902200
C	1.92897900	0.15907900	-0.94567900
C	2.58975800	-0.46050700	0.12570100
C	1.80200500	-0.99025200	1.16000800
C	0.41719400	-0.88042000	1.14093500
C	4.07434400	-0.61366700	0.14534700
C	4.91907000	0.42797500	-0.08982000
C	4.55280600	-1.99618200	0.45362100
C	6.38522700	0.24672000	-0.30567200
C	4.44962000	1.84635600	-0.13611400
C	4.04655700	-3.10004200	-0.24910400
C	4.47599100	-4.39176100	0.04327100
C	5.40371500	-4.60800800	1.06169100
C	5.89878400	-3.52221800	1.78213500
C	5.47953400	-2.22932200	1.47951700
C	7.30843000	1.05186700	0.37903900
C	8.67674600	0.90185000	0.17276000
C	9.14865100	-0.03772100	-0.74309500
C	8.24146300	-0.82674200	-1.44866200
C	6.87375700	-0.68887800	-1.22932000
C	4.79694900	2.67705300	-1.21248600
C	4.38649600	4.00645400	-1.25011700
C	3.64512300	4.54205400	-0.19660300
C	3.31500000	3.73518500	0.89132400
C	3.70966000	2.39932300	0.91874700
H	-0.96966000	-2.75230800	-0.10386400
H	-0.53994800	2.35291500	0.25196000
H	-4.16541400	6.79748800	-0.61780800
H	-3.61798900	6.60781000	1.04686000
H	-1.64948900	6.94632700	-0.32031600
H	-2.16169100	5.74618200	-1.50423600
H	-1.41863400	5.17525800	1.41149800
H	-0.44645700	4.73547000	0.01495600
H	-2.08899000	-5.42511700	0.86281300
H	-1.32016900	-5.07578800	-0.67505200
H	-2.88403900	-7.09311000	-0.74942000
H	-3.34107500	-5.83792000	-1.89960000
H	-5.34302800	-6.58639600	-0.69195500
H	-4.56330300	-6.32375700	0.86725600
H	-7.04916300	-5.30386100	-0.47882600
H	-6.83186800	-4.42860200	1.03628300
H	-8.14020100	-3.04978700	-0.54514100
H	-6.89365200	-3.22213200	-1.77801900
H	-6.68419800	-1.73138900	0.88621400
H	-6.53403300	-1.05227900	-0.71904800
H	-6.30853700	2.44722600	-0.98091600
H	-6.34878400	1.60207300	0.55319700
H	-7.53553500	3.85548700	0.62371700
H	-6.23475600	3.72229400	1.80379800
H	-6.03409100	5.88004000	0.68393200
H	-6.12882900	5.13561100	-0.91125800
H	0.06199600	0.74708700	-1.81739700
H	2.50809100	0.55034200	-1.77271700
H	2.28384900	-1.48814900	1.99340100

H	-0.16263300	-1.27727400	1.96681400
H	3.32243900	-2.94225900	-1.04125700
H	4.08865600	-5.23020000	-0.52511400
H	5.73597600	-5.61355400	1.29299000
H	6.61433300	-3.68104400	2.58100000
H	5.87388000	-1.39072300	2.04078900
H	6.95084900	1.79546600	1.08239200
H	9.37477300	1.52315000	0.72249000
H	10.21380500	-0.14833400	-0.91108400
H	8.59885100	-1.54997000	-2.17301300
H	6.17653000	-1.30691000	-1.78193600
H	5.39402700	2.27545800	-2.02348200
H	4.65851900	4.62922100	-2.09497900
H	3.34663700	5.58430700	-0.21545800
H	2.76522400	4.14979200	1.72949300
H	3.46021500	1.78152200	1.77349300

Table S1 Photophysical Properties of the TPEF in Various Solvents

Solvent	Absorption Peak (nm)	Emission Peak (nm)	Stokes' Shift (nm)
Ethanol	580.0	604.0	24.0
MeCN	580.0	606.0	26.0
Methanol	579.0	603.0	24.0
Acetone	581.0	607.0	26.0
CHCl3	580.0	600.0	20.0
Ethyl Estate	581.0	605.0	24.0
CH2Cl2	580.0	601.0	21.0
THF	583.0	607.0	24.0

Calculation 2:

Computational UV Calculations for TPEF:

- Calculation Type: FREQ
- Calculation Method: RB3LYP TD-FC
- Basis Set: 6-311+G(d,p)
- Charge: 1
- Spin: Singlet
- Solvation: scrf=(cpcm,solvent=methanol)
- E(TD-HF/TD-DFT): -2155.6077 Hartree
- RMS Gradient Norm: 3.33e-06 Hartree/Bohr
- Imaginary Freq: 0
- Dipole Moment: 17.483934 Debye
- Polarizability (α): 1221.9903 a.u.
- **Calculated wavelength**: 565.97 nm

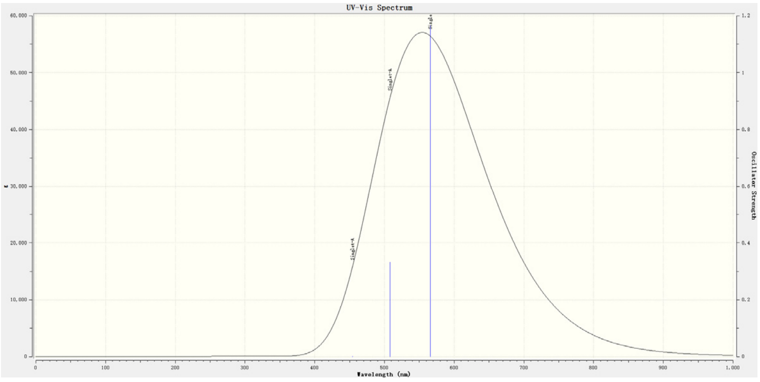

Figure S1 Calculated UV of TPEF in methanol from Calculation 2

The coordinates of the optimized structures

C	1.68776300	0.07448300	0.16710100
C	2.43258000	1.28725300	-0.01301900
C	3.84945000	1.24630300	-0.09728800
O	4.52628800	0.06083000	0.01955400
C	3.85452400	-1.11654700	0.21868300
C	2.43710400	-1.14604200	0.28200700
C	1.85177300	2.58293300	-0.06310300
C	2.58787700	3.73394200	-0.22342700
C	4.01538900	3.64399900	-0.35799500
C	4.64574800	2.36579400	-0.28769200
C	4.65472000	-2.24418400	0.32768300
C	4.02258100	-3.51505700	0.48186500
C	2.58929000	-3.59092100	0.51831800
C	1.85167800	-2.43533500	0.40600000
N	4.77403600	-4.64908500	0.57012000
C	4.17792800	-5.98970600	0.61249100
C	2.77662700	-6.02425700	0.02071300
C	1.92198800	-4.93465900	0.66694200
C	1.91413900	5.08478700	-0.23128600
C	2.72932700	6.09405900	-1.03678000
C	4.16403100	6.10946700	-0.52945400
N	4.76484000	4.77251900	-0.54276000
C	6.22773200	4.72349700	-0.49685400
C	6.75841400	3.43499000	-1.10830700
C	6.14496500	2.22695200	-0.40331800
C	6.15825100	-2.14497500	0.28557000
C	6.78281000	-3.28865100	1.08405800
C	6.23981000	-4.62280200	0.59880100
C	0.21702800	0.08115600	0.23760700
C	-0.56299800	0.64563000	-0.78966800
C	-1.95061300	0.64649600	-0.72472800
C	-2.62968700	0.09911700	0.37524700
C	-1.85297700	-0.43875200	1.41471000
C	-0.46643400	-0.46530200	1.34116000
C	-4.11698500	0.13701000	0.47359300
C	-4.93438700	-0.26711000	-0.53765700
C	-4.64571800	0.65153800	1.77497000
C	-6.40446600	0.00149000	-0.54090500
C	-4.43248400	-1.01935400	-1.72767300
C	-4.19068700	1.87138400	2.29874100
C	-4.66449400	2.34741300	3.51919900
C	-5.58661400	1.60028400	4.25374600
C	-6.02997900	0.37492300	3.75577600

C	-5.56537400	-0.09303600	2.52788700
C	-7.31910500	-1.03355800	-0.79157800
C	-8.69010400	-0.78808400	-0.81296400
C	-9.17534800	0.50453500	-0.60836700
C	-8.27655000	1.54726700	-0.38223700
C	-6.90602500	1.29739400	-0.34703600
C	-4.75966000	-0.60273000	-3.02754800
C	-4.31181100	-1.31113300	-4.14027200
C	-3.54717300	-2.46709100	-3.97560000
C	-3.23457000	-2.90599600	-2.68868900
C	-3.67091200	-2.18789500	-1.57696600
H	0.77912800	2.67239300	0.04539200
H	0.77275500	-2.51460100	0.40600800
H	4.84803400	-6.65922800	0.06695000
H	4.16221600	-6.33135200	1.65722300
H	2.34678400	-7.01368500	0.19003200
H	2.82875600	-5.86821700	-1.06153200
H	1.79057400	-5.16381300	1.73275000
H	0.92257500	-4.90253300	0.22765400
H	1.81273500	5.44520000	0.80090000
H	0.90115300	4.98946700	-0.62866300
H	2.30957000	7.09790200	-0.94406300
H	2.71919300	5.82905400	-2.09877100
H	4.79114200	6.75406400	-1.14835000
H	4.20079300	6.50864100	0.49554300
H	6.60251600	5.59597800	-1.03380000
H	6.55909300	4.81587900	0.54856600
H	7.84679900	3.41822000	-1.02101600
H	6.51394700	3.41471700	-2.17484600
H	6.57745400	2.12331500	0.60030400
H	6.39030400	1.30629200	-0.93512900
H	6.50310500	-2.18753800	-0.75603200
H	6.47867900	-1.17899200	0.67662300
H	7.86938800	-3.28831900	0.97657200
H	6.55782700	-3.16583700	2.14815500
H	6.56789600	-5.43762300	1.24967400
H	6.61282200	-4.84751500	-0.41085400
H	-0.07471000	1.07233000	-1.65837400
H	-2.51788300	1.08266800	-1.53813300
H	-2.34258300	-0.84607700	2.29217200
H	0.09875400	-0.88801000	2.16397900
H	-3.46836500	2.45587600	1.73967900
H	-4.31146000	3.30000700	3.89860400
H	-5.95013400	1.96632200	5.20731900
H	-6.73540300	-0.22016700	4.32524600
H	-5.91234100	-1.04757400	2.15015900
H	-6.95274900	-2.04011800	-0.96014800
H	-9.37982300	-1.60529500	-0.99309600
H	-10.24184200	0.69790000	-0.63281300
H	-8.64187000	2.55804100	-0.23741400
H	-6.21543800	2.11443100	-0.17533200
H	-5.36102900	0.28881300	-3.16663200
H	-4.56335700	-0.96317600	-5.13609700
H	-3.20558500	-3.02382200	-4.84092600
H	-2.65450200	-3.81161400	-2.54921300
H	-3.42759700	-2.53858600	-0.58094100

Calculation 3

- Calculation Method: RB3LYP TD-FC
- Basis Set: 6-311+G(d,p)
- Spin: Singlet
- Solvation: scrf=solvent=methanol
- E(TD-HF/TD-DFT): -2155.6073 Hartree
- RMS Gradient Norm: 2.239e-06 Hartree/Bohr
- Imaginary Freq: 0
- Dipole Moment: 17.442659 Debye
- Polarizability (α): 1216.4905 a.u.
- **Calculated wavelength**: 565.17 nm

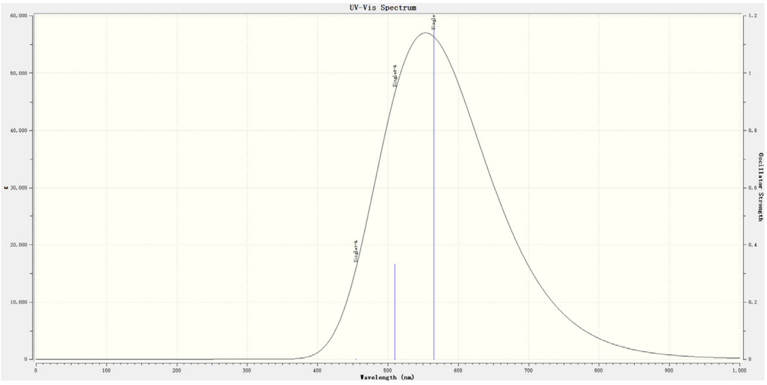

Figure S2 Calculated UV of TPEF in methanol from Calculation 3

The coordinates of the optimized structures

C	1.68771800	0.07331600	0.16754400
C	2.43148600	1.28711600	-0.01052900
C	3.84831300	1.24754400	-0.09545600
O	4.52629000	0.06263100	0.01973200
C	3.85572200	-1.11561300	0.21723900
C	2.43838100	-1.14669600	0.28022300
C	1.84964500	2.58242400	-0.05782300
C	2.58469000	3.73422900	-0.21704800
C	4.01210100	3.64564400	-0.35334000
C	4.64360000	2.36802100	-0.28486900
C	4.65712900	-2.24261600	0.32488000
C	4.02630800	-3.51425100	0.47731400
C	2.59309200	-3.59186400	0.51181300
C	1.85432300	-2.43686100	0.40108400
N	4.77904100	-4.64755200	0.56555600
C	4.18403700	-5.98861600	0.60737600
C	2.78451300	-6.02460900	0.01148200
C	1.92676100	-4.93661600	0.65624500
C	1.90996100	5.08457500	-0.22184700
C	2.72287200	6.09530700	-1.02779300
C	4.15846700	6.11149500	-0.52301200
N	4.76037800	4.77508400	-0.53785200
C	6.22330800	4.72737600	-0.49322100
C	6.75472500	3.43980600	-1.10606000
C	6.14285600	2.23061900	-0.40159500
C	6.16056200	-2.14167500	0.28315000
C	6.78631500	-3.28529800	1.08077500
C	6.24472000	-4.61971600	0.59458200
C	0.21717600	0.07848400	0.23807800
C	-0.56338900	0.64653500	-0.78689300

C	-1.95091200	0.64646800	-0.72178300
C	-2.62975300	0.09458900	0.37614700
C	-1.85250300	-0.44719700	1.41324700
C	-0.46600400	-0.47288200	1.33942300
C	-4.11699500	0.13166900	0.47521700
C	-4.93486900	-0.26466200	-0.53897800
C	-4.64526300	0.63609100	1.78074000
C	-6.40478800	0.00464600	-0.54026500
C	-4.43353900	-1.00838000	-1.73456700
C	-4.18772300	1.85029100	2.31534000
C	-4.66088200	2.31671300	3.53971700
C	-5.58495200	1.56534400	4.26738000
C	-6.03097000	0.34546800	3.75852100
C	-5.56699100	-0.11278500	2.52681100
C	-7.31960300	-1.02721100	-0.80319600
C	-8.69046000	-0.78106300	-0.82317100
C	-9.17546200	0.50934900	-0.60480700
C	-8.27653500	1.54914600	-0.36623400
C	-6.90617900	1.29845600	-0.33251900
C	-4.76290700	-0.58349100	-3.03120900
C	-4.31571100	-1.28385800	-4.14922600
C	-3.54956500	-2.43999100	-3.99333900
C	-3.23482100	-2.88715300	-2.70982800
C	-3.67046100	-2.17703500	-1.59277500
H	0.77708100	2.67096700	0.05217300
H	0.77549900	-2.51736900	0.39939600
H	4.85633400	-6.65810400	0.06452100
H	4.16523100	-6.32940500	1.65238300
H	2.35538300	-7.01469900	0.17878800
H	2.83958600	-5.86765500	-1.07048200
H	1.79188500	-5.16754500	1.72122800
H	0.92881500	-4.90486900	0.21357600
H	1.81037400	5.44366100	0.81099200
H	0.89626000	4.98904300	-0.61734300
H	2.30244000	7.09872600	-0.93349000
H	2.71104900	5.83123900	-2.09000200
H	4.78393100	6.75683200	-1.14284300
H	4.19676300	6.51037500	0.50205600
H	6.59691900	5.60066300	-1.02971700
H	6.55549300	4.81929300	0.55200000
H	7.84320600	3.42394700	-1.01966400
H	6.50944500	3.42014200	-2.17242000
H	6.57618300	2.12637800	0.60163000
H	6.38863700	1.31062300	-0.93436100
H	6.50569000	-2.18294200	-0.75842900
H	6.47981300	-1.17562400	0.67501100
H	7.87290700	-3.28378000	0.97333000
H	6.56125200	-3.16348600	2.14497700
H	6.57375200	-5.43451200	1.24502000
H	6.61809100	-4.84347800	-0.41519700
H	-0.07544500	1.07644900	-1.65420400
H	-2.51836000	1.08522300	-1.53364800
H	-2.34176900	-0.85788600	2.28932000
H	0.09946600	-0.89846100	2.16057200
H	-3.46367100	2.43788200	1.76179400
H	-4.30582800	3.26514300	3.92759100
H	-5.94809000	1.92392400	5.22393200
H	-6.73821400	-0.25284300	4.32233000
H	-5.91635800	-1.06297100	2.14046300
H	-6.95342900	-2.03188800	-0.98294600

H	-9.38025500	-1.59600000	-1.01310300
H	-10.24187700	0.70334500	-0.62810100
H	-8.64165500	2.55835400	-0.21037300
H	-6.21559500	2.11331500	-0.15083600
H	-5.36587200	0.30799600	-3.16351700
H	-4.56913100	-0.92959600	-5.14235600
H	-3.20852800	-2.99056300	-4.86282100
H	-2.65358300	-3.79306300	-2.57722900
H	-3.42534600	-2.53406000	-0.59944800

（2）TPEF Staining Microplastic Particles

The microplastic particles used in this study were obtained from Dong Guan Ming Yu Xing Plastic Starting Materials Ltd. These particles consisted of polyvinyl chloride (PVC), polystyrene (PS), polymethyl methacrylate (PMMA), and polyethylene (PE). Each type of particle had a size, with PVC, PS and PMMA measuring 1 μm and PE measuring 5 μm. These particles were used in their form without any treatment or modifications except for the staining process explained in the manuscript.

Subsequent to the staining process, the stained microplastics were initially suspended in water before being meticulously transferred onto glass slides for ensuing qualitative analysis. Observations were conducted using a fluorescence microscope equipped with an L208F-3M830F assisted 8.3 million-pixel electronic eyepiece. Imaging was executed with an AOSVI L208F-3M830F light microscope, utilizing a light-emitting diode (LED) as the fluorescence source. A green light spectrum, ranging from 495 nm to 555 nm, was employed for the excitation of the stained microplastic particles.

The outcomes of the staining, as illustrated in Figure S5, revealed a pronounced disparity in fluorescence intensity across the various types of microplastics. A robust fluorescent dyeing effect was discerned in PS particles across three different sizes, as well as in PVC and PMMA particles. Conversely, PE particles manifested a relatively subdued fluorescence intensity under identical experimental conditions.(Figure S3)

It is crucial to emphasize that particles devoid of TPEF staining remained undetectable under the conditions specified, thereby accentuating the efficacy of TPEF as a qualitative staining agent.under the specified conditions, which underscores the effectiveness of TPEF as a staining agent.

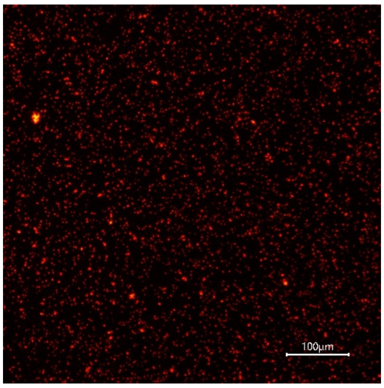

(a)

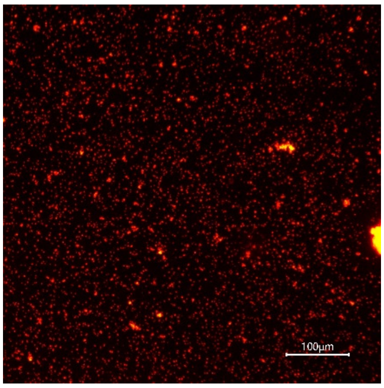

(b)

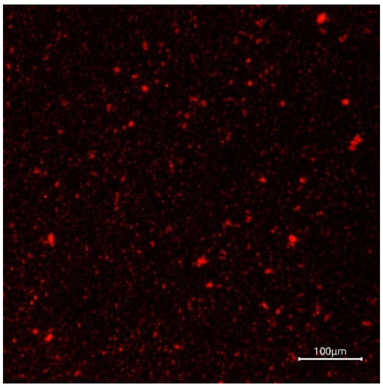

(c)

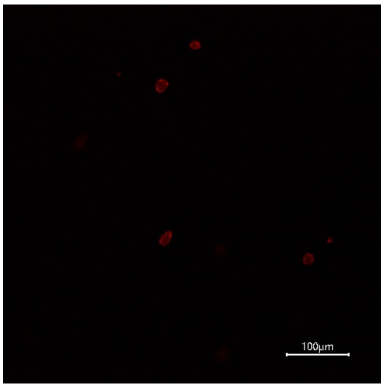

(d)

Figure S3: TPEF dyed microplastic particles(a) PVC(1μm); (b) PMMA(1μm); (c) PS(1μm); (d) PE(5μm).

(3) TEM and SEM images of PS nanoparticles

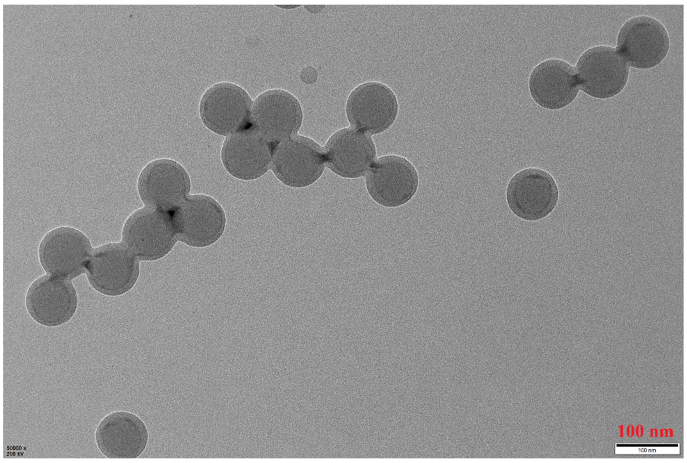

(a)

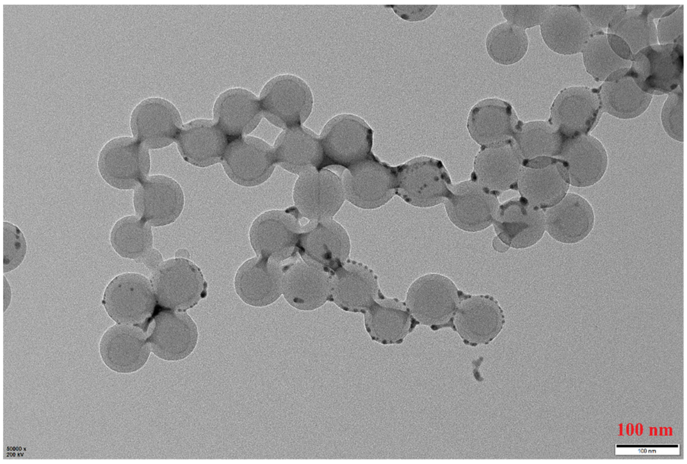

(b)

Figure S4 TEM images of PS nanoparticles. (a) Before TPEF staining; (b) After TPEF staining.

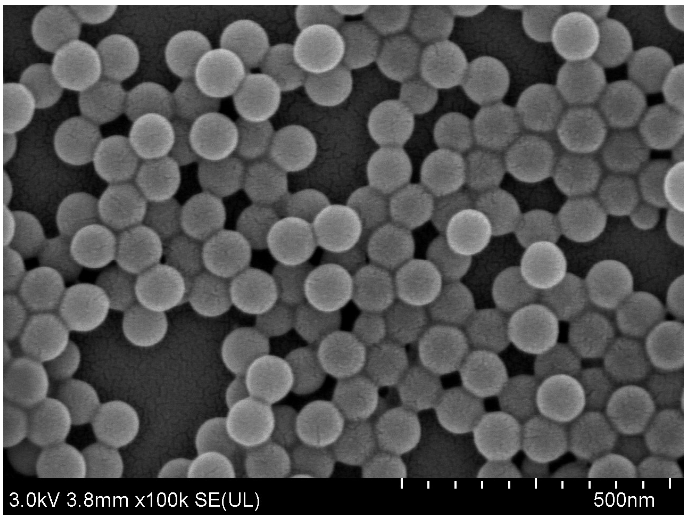

(a)

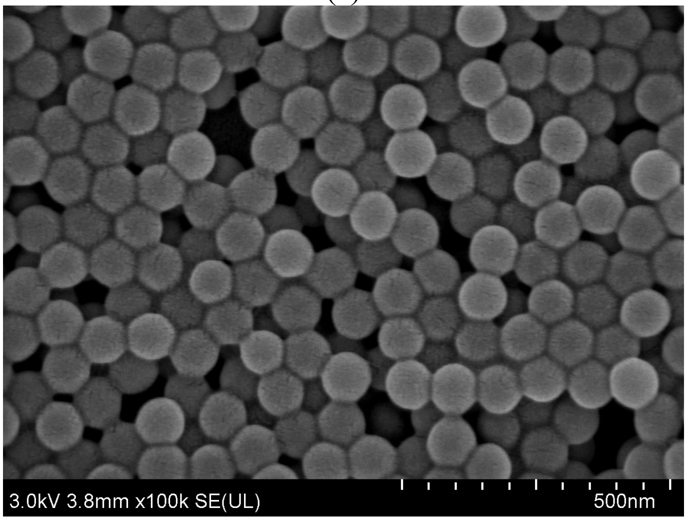

(b)

Figure S5 SEM images of PS nanoparticles. (a) Before TPEF staining; (b) After TPEF staining.

(4) Nanoplastic Particles in Bean Sprouts or Hela Cells

Table S2. CLSM images of control mung bean sprouts and soybean sprouts samples.

Position	hypocotyl epidermis		cross-sections of hypocotyls	
Particles / Time (Day)	Group M-water	Group M- PS	Group M-water	Group M- PS
Mung Bean Day 1	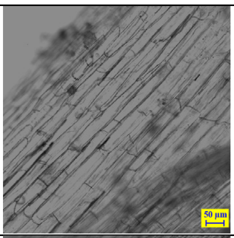	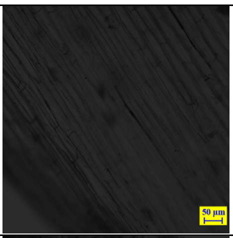	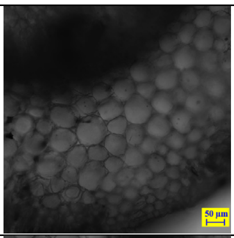	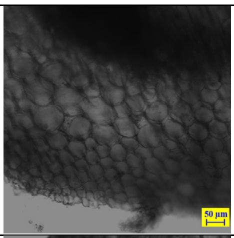
Mung Bean Day 3	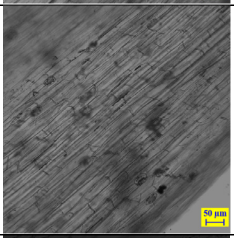	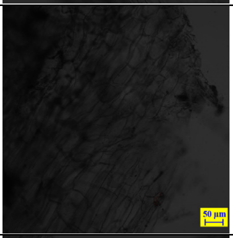	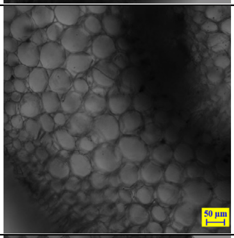	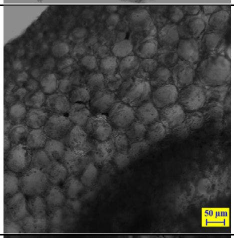
Mung Bean Day 5	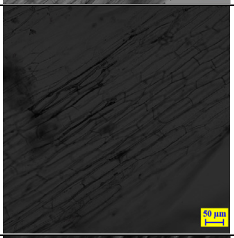	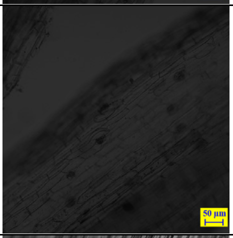	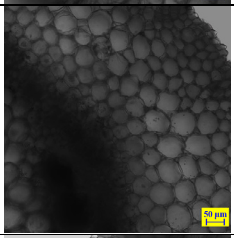	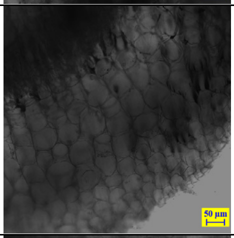
Soybean Day 1	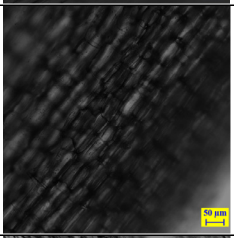	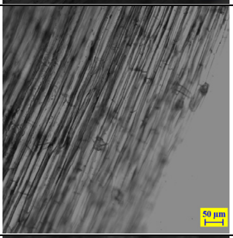	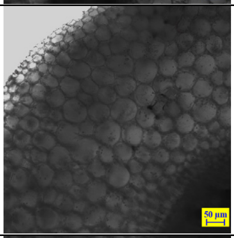	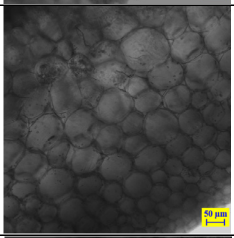
Soybean Day 3	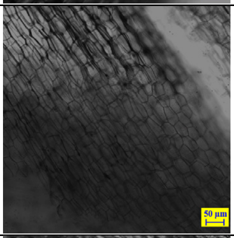	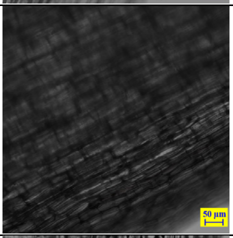	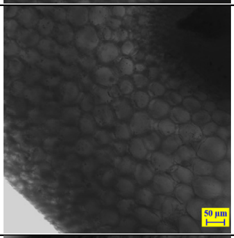	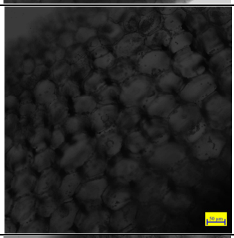
Soybean Day 5	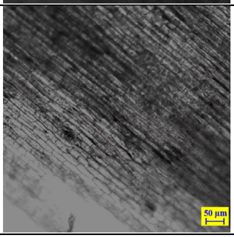	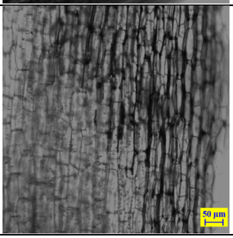	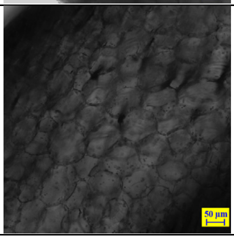	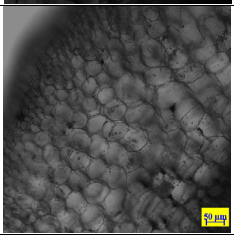

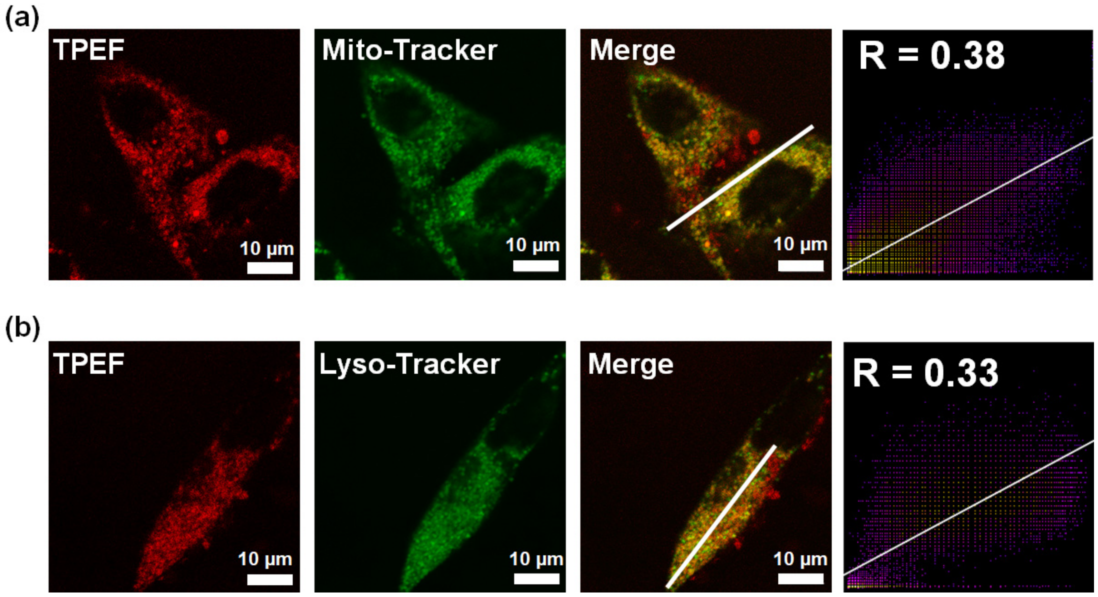

Figure.S6 Confocal images of live HeLa cells incubated with TPEF, (100 μg/mL, λex: 561 nm, λem: 580-650 nm) and co-stained with (a) Mito-Tracker Green dye (1 μM, λex:488 nm, λem: 500-550 nm) or (b) Lyso-Tracker Green dye (1 μM, λex:488 nm, λem: 500-550 nm). Scale bar: 10 μm.

(5) Spectra to TPE-CHO and TPEF

^{1}H , ^{13}C NMR Spectra

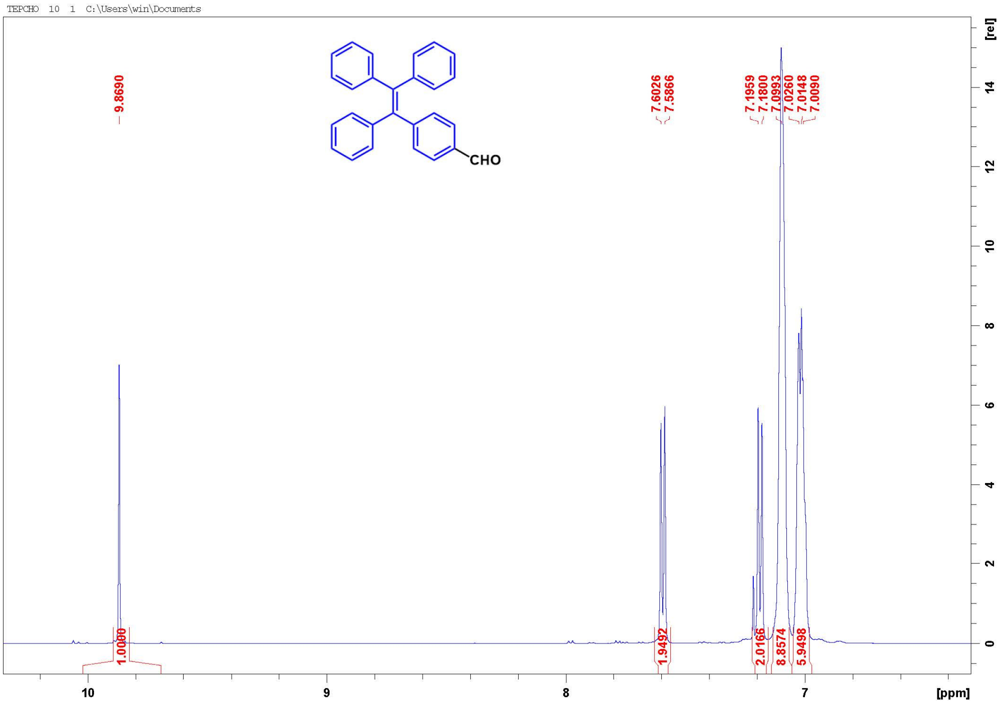

Figure.S7 ^{1}H NMR of intermediate product TPE-CHO.

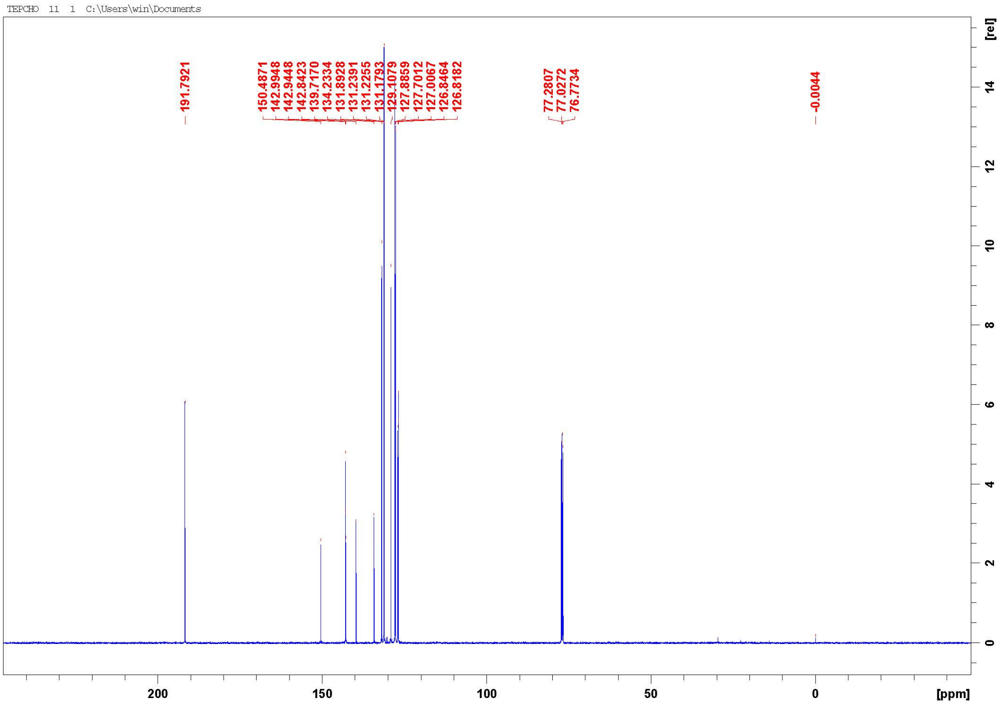

Figure.S8 ^{13}C NMR of intermediate product TPE-CHO.

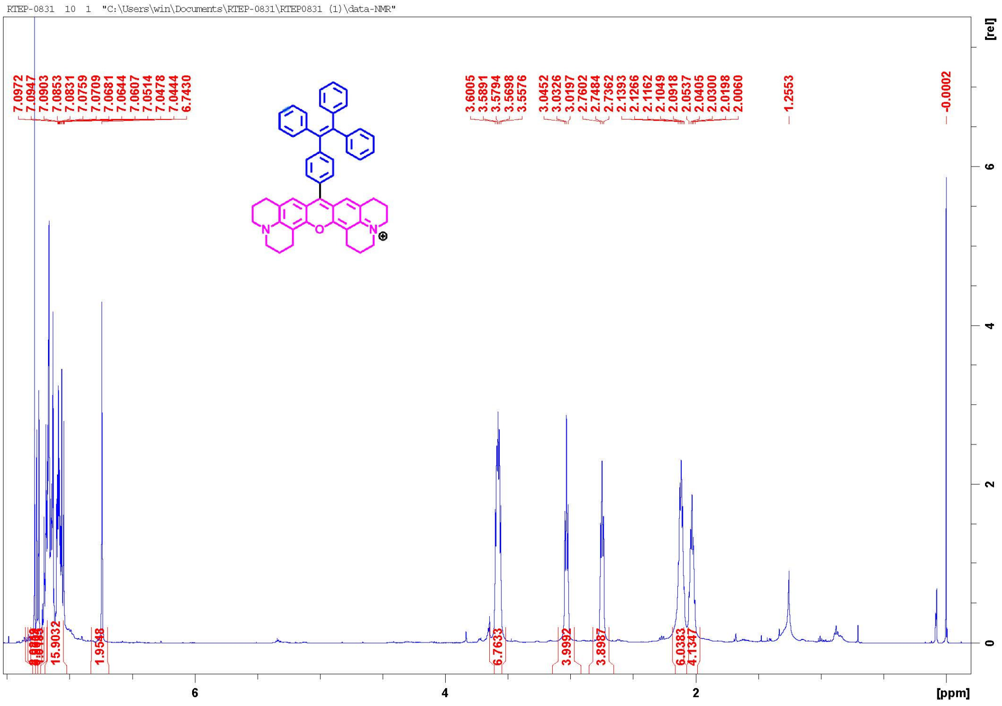

Figure.S9 ^{1}H NMR of intermediate product TPEF.

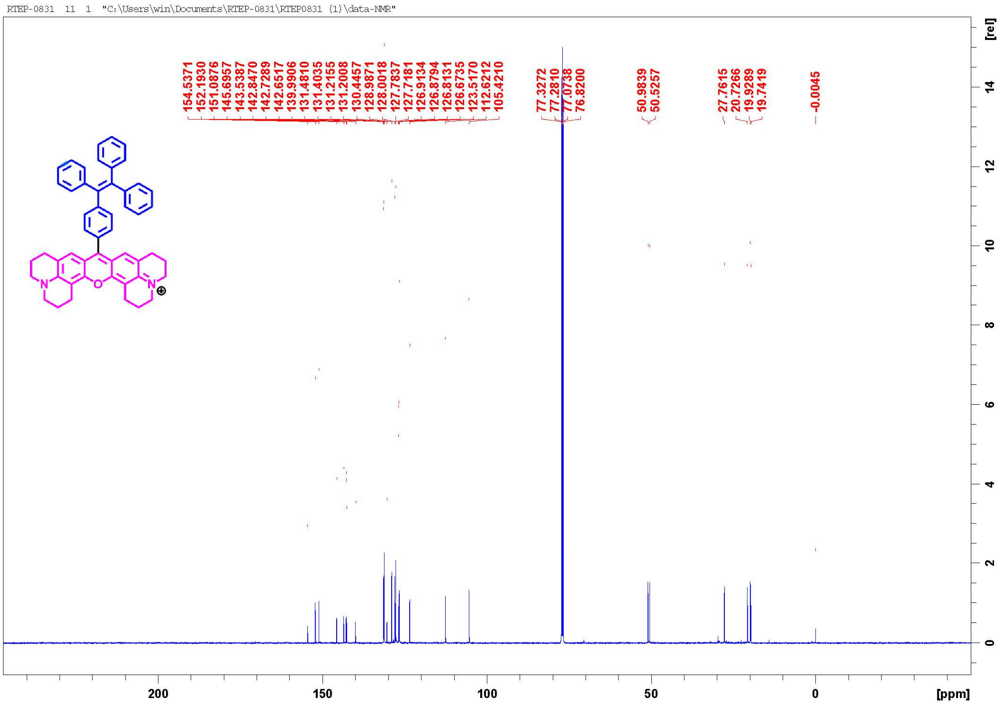

Figure.S10 ^{13}C NMR of intermediate product TPEF.

National Center for Organic Mass Spectrometry in Shanghai
Shanghai Institute of Organic Chemistry
Chinese Academic of Sciences
High Resolution ESI-MS REPORT

Instrument: Thermo Scientific Q Exactive HF Orbitrap-FTMS

Card Serial Number: E221137

Sample Serial Number: RTEP

Operator: Songw Date: 2022/07/12

Operation Mode: ESI Positive Ion Mode

Elemental composition search on mass 701.3525

m/z= 696.3525-706.3525

m/z	Theo. Mass	Delta (ppm)	RDB equiv.	Composition
701.3525	701.3526	-0.14	30.5	$C_{51}H_{45}ON_2$

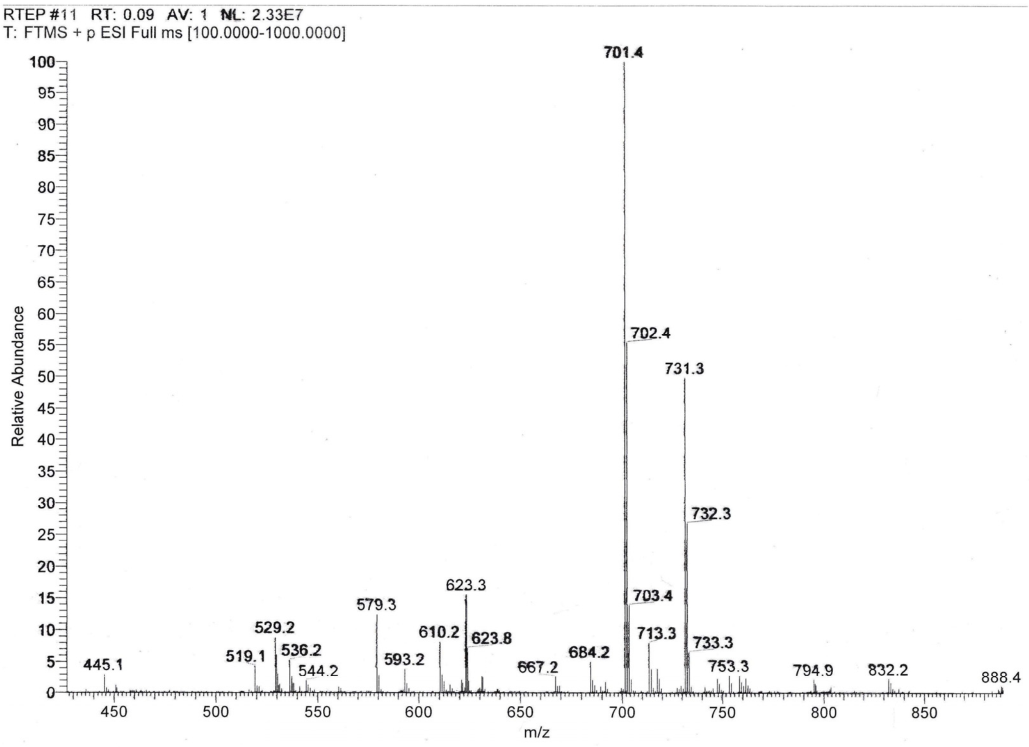

Figure.S11 HRMS of TPEF.
